# Smart and Flexible Optical Solar Reflectors for Passive Radiative Cooling Regulation in Space Using a W:VO_2_ Metasurface

**DOI:** 10.1002/nap2.70011

**Published:** 2026-01-28

**Authors:** Mirko Simeoni, Kai Sun, Alessandro Urbani, Ioannis Zeimpekis, Ilja Czolkos, Lars Kildebro, Matteo Gaspari, Giovanni Bartolini, Behcet Alpat, Jiri Frolec, Tomas Kralik, Cornelis H. (Kees) de Groot, Otto L. Muskens, Sandro Mengali

**Affiliations:** ^1^ Consorzio C.R.E.O. L'Aquila Italy; ^2^ Physics and Astronomy, Faculty of Engineering and Physical Sciences University of Southampton Southampton UK; ^3^ Electronics and Computer Science, Faculty of Engineering and Physical Sciences University of Southampton Southampton UK; ^4^ NIL Technology Kongens Lyngby Denmark; ^5^ BEAMIDE S.r.l Perugia Italy; ^6^ Institute of Scientific Instruments of the CAS Brno Czech Republic

**Keywords:** metasurface, optical solar reflector, radiative cooling, thermal coating, vanadium dioxide

## Abstract

Smart optical solar reflectors (OSRs) with temperature‐adaptive radiative emission around room temperature are highly desirable for passive thermal management in spacecraft. This work demonstrates a smart and flexible metasurface‐based OSR, or meta‐OSR, consisting of an optimized W:VO_2_‐based metasurface and a low emissivity solar reflector. The fabricated smart meta‐OSR overcomes long‐standing challenges by combining a solar absorption (*α*) of 0.22, high‐temperature emissivity (*ε*_hot_) of 0.8, infrared emissivity contrast (Δ*ε*) of 0.33 and a transition temperature (*T*_MIT_) of 30°C. In addition, through the use of nanoimprint lithography and a low‐temperature W:VO_2_ process, the smart meta‐OSR is demonstrated over an area of 10 × 10 cm^2^ on space‐grade polyimide, achieving significant weight reduction and easy integration on satellite surfaces. The fabricated devices successfully passed various space qualification tests, including thermal cycling, proton and electron radiation, adhesion, bending and humidity resistance, showing negligible performance degradation. The smart meta‐OSRs in this work are production‐ready and hold promise as next‐generation thermal control solution for ultralight spacecraft and small satellites.

## Graphical abstract

Passive regulation of thermal management in space relies on engineered radiative coatings that can vary their emissivity with temperature. Here a smart and flexible solution is presented combining a W:VO_2_ metasurface with a low‐emissivity solar reflector. This highly durable first‐surface coating survives space qualification tests and offers attractive performance metrics as light‐weight and flexible solution for space‐based and terrestrial applications.

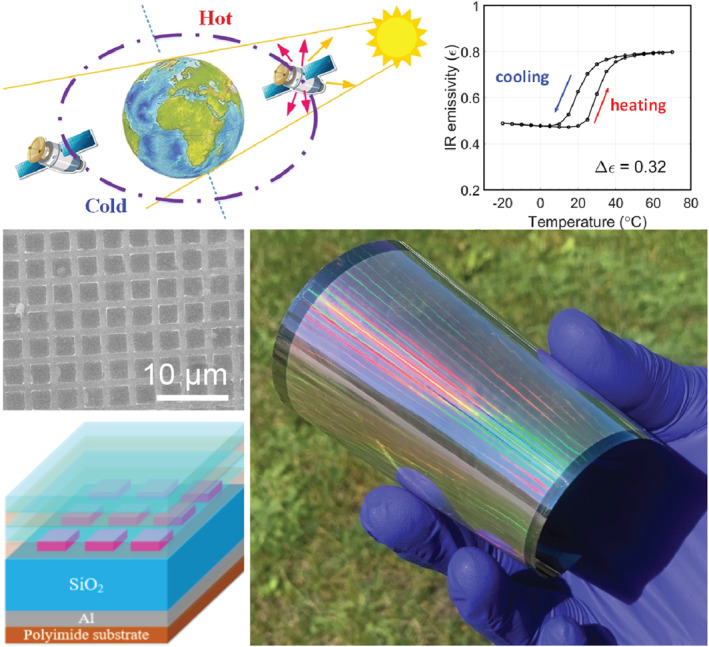

## 1 Introduction

Thermal management of spacecraft and satellites is required to maintain the internal temperature within the operational range of electronic components and system. Optical solar reflectors (OSRs) are vital components in space technology, used to manage the extreme thermal conditions by reflecting solar radiation and radiating away excess heat [1]. Conventional OSRs typically have a high reflection of solar radiation (low solar absorption, α) and high emission of infrared (IR) radiation at 300 K (IR emissivity, *ε*) and are consists of aluminium plated glass tiles with a thickness of about 50 μm for weight consideration. However, these ultra‐thin glass tiles are fragile and require specialized handling during satellite assembly, integration and testing. Recent years have seen advances in the development of novel first‐surface solutions based on thin film and metasurface coatings on flexible substrates which could offer lightweight solutions [2, 3], of particular interest for small satellites and future constellations for space‐based solar power and orbital data centers [4].

Although the conventional static OSRs provide optimized cooling performance during operation, the continuous high emissivity provides a constant high thermal dissipation even when the spacecraft is below its optimal temperature, for example during an eclipse, interplanetary travel, orbit insertion or power down phases. The spacecraft compensates this by heating, requiring additional electricity consumption from the onboard battery and involving additional mass and complexity of the thermal control and power distribution systems. Therefore, smart thermal control systems with tunable emissivity are highly desirable. Smart thermal control systems are divided into two categories, active and passive systems. Active thermal control normally involves a sensor, control system and moving parts such as louvres and thus has a high system complexity, power consumption and risk of failure [5, 6, 7]. In contrast, passive thermal control is achieved through the tunability of materials with varying emissivity, such as thermochromics based on perovskite manganese oxide [3], vanadium dioxide (VO_2_) [8], electrochromic metal–organic polymer [9] and shape‐memory materials [10].

Compared with organic materials, VO_2_ is promising for its durability against harsh space environments, atomic oxygen corrosion and high ion and electron flux [3]. The thermochromic properties of undoped VO_2_ are governed by an insulator–metal‐transition at a critical temperature of around 68°C [11, 12], which can be reduced by doping of VO_2_ with elements such as W, Mg, Ta, Al and Ti [13, 14, 15, 16]. A number of proof‐of‐principle studies have been reported for the tunable emissivity OSR based on VO_2_ and a high emissivity contrast (Δ*ε*) of 0.5 could be achieved experimentally [17, 18, 19]. Because of the high solar absorption of VO_2_, most works reported *α* around 0.5, far beyond the accepted value for OSRs of about 0.2 [8, 20, 21]. Recent efforts, including our previously reported VO_2_ metasurface solution, have managed to improve *α* to around 0.3 by structuring the material [8, 19, 22, 23]. However, these values for *α* are still too high. Another point of consideration is that pure VO_2_ has a high transition temperature at around 68°C, which is incompatible with the optimized operating temperature of electrical systems [23]. In addition, most demonstrations required a high temperature annealing step above 400°C for VO_2_ film formation, which is incompatible with space grade polyimide substrates, and therefore silicon substrates were used. Last, as a general challenge for metasurfaces, small‐area demonstrations using photolithography or e‐beam lithography are incompatible with or too expensive for scaling up. Recent work [24] proposed a W:VO_2_‐based terrestrial radiative cooling solution that addresses several of the challenges above but with some critical limitations in the fabrication process.

For next generation smart OSRs to be attractive and widely adopted, critical improvements need to be addressed, achieving (i) reduced solar absorptance around 0.2, (ii) phase transition around room temperature, (iii) compatibility with space grade flexible substrates and (iv) scalable manufacturing. Here, we demonstrate a novel smart metasurface‐based optical solar reflector (meta‐OSR) to address all these four critical improvements of VO_2_ smart OSRs. The novel smart meta‐OSRs are fabricated over a large area of 10 × 10 cm^2^ and are formed on polyimide foil using Nanoimprint lithography technology. A low emissivity solar reflector (LESR) is integrated onto the structure to selectively reflect solar radiation for solar absorptance improvement. Through a tungsten doping, the transition temperature is reduced to be around 30°C. The fabricated smart meta‐OSRs successfully passed a ground test campaign equivalent to 2 years in the GEO environment bringing the technology to preliminary validation for the space‐relevant environment. Smart meta‐OSRs can offer a high advantage in space applications as lightweight technology with low launch cost and easy assembly on curved surfaces.

## 2 Smart Meta‐OSR Design and Fabrication

**FIGURE 1:**
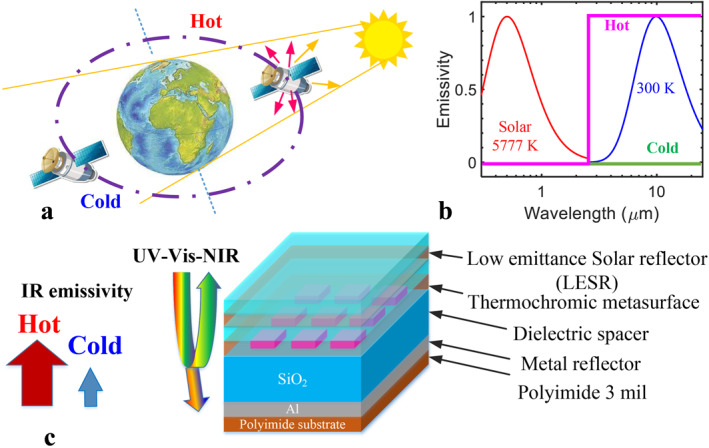
Smart W:VO_2_ metasurface optical solar reflectors with tunable emissivity. (a) Satellite orbiting around the earth facing and eclipsed from the sun, (b) ideal optical response of the smart meta‐OSR at high and low temperatures (hot and cold), and (c) meta‐OSR schematic with low emissivity solar reflector (LESR) cap.

The operation mechanism of the proposed smart meta‐OSR is schematically shown in Figure 1a. When a satellite orbits around the earth, it can be either facing the sun under solar radiation or eclipsed by the earth without solar radiation exposure. In terms of thermal management when facing the sun, the satellite surface is desired to reflect the solar radiation and achieve radiative cooling through its own black‐body radiation. When the satellite is eclipsed by the earth, the radiative cooling from the satellite surface becomes undesirable as electrical heating from battery power would be required to maintain a suitable temperature for electrical system operation. Therefore, the desirable or targeted OSR optical response can be presented in Figure 1b. At elevated temperatures above 30°C, the OSR requires a lower absorption (emissivity) in the solar radiation band (0.3–2.5 μm) and a high emissivity in the IR band (3–30 μm). The curves in Figure 1b represent the normalized blackbody spectra at 5777 and 300 K, respectively [25]. At temperatures below 30°C, low IR emissivity is desired while the solar absorption is irrelevant.

To address these requirements, a novel smart meta‐OSR design is proposed as Figure 1c. The main OSR stack is made of a perfect metamaterial absorber (PMA) stack [2, 26] on polyimide substrate with a LESR on top. The PMA consists of a back‐reflector, a SiO_2_ spacer and a W:VO_2_ metasurface, which is formed by patterned W:VO_2_ square features with gap in between. The SiO_2_ spacer thickness is optimized for a strong absorption in IR range when W:VO_2_ is in the metallic phase, labelled here as ‘hot’. The composition of the W:VO_2_ thermochromic material is chosen for its transition near room‐temperature [27]. The LESR stack consists of a superposition of several distributed Bragg reflectors (DBRs) designed by alternating high and low refractive index materials with a total thickness of 2–2.5 μm, aiming to achieve a high reflection band in the visible spectral range 400–750 nm. The LESR reduces the interaction of the incoming visible light with the W:VO_2_ layer alleviating the high solar absorption associated with this material.

In this work we used W:VO_2_ materials fabricated through two different methods. Atomic layer deposition (ALD) was developed at University of Southampton, whereas sputtering followed by oxidation was developed at Consorzio C.R.E.O., with details described in Experimental Section. Both developed materials performed very similarly as described in the next section. The ALD‐grown W:VO_2_ was used for small‐area devices on a silicon substrate to explore the design variations of the meta‐OSR stack without LESR. The large‐area final demonstrator devices with LESR were obtained using sputtering of metallic V and W followed by a thermal oxidation step. All layers in the final demonstrator devices are formed on a flexible polyimide substrate to meet the goal of easy assembly and low launch cost.

## 3 Results

### 3.1 Characterization of Unpatterned Thin‐Film W:VO_2_ OSRs

For the small‐area and large‐area devices, W:VO_2_ films were deposited on the SiO_2_ spacer–Al reflector stack. The unpatterned layers were subsequently characterized using Raman spectroscopy and were confirmed to be monoclinic W:VO_2_ (Supporting Information S1: Supplementary Materials S1). Their infrared responses were characterized using Fourier Transform Infrared Spectroscopy (FTIR) with heating stage and IR emissivity as normal emissivity, was calculated from the spectral response (Supporting Information S1: Supplementary Materials S2 and S3). Supporting Information S1: Figure S4 (Supplementary Materials S4) shows the IR absorption spectra against the temperature for the heating part of the cycle and the emissivity hysteresis (*ε*) over the full cycle. Good IR absorption contrasts are achieved for both ALD and sputtered W:VO_2_ thin‐film reflectors with an IR emissivity contrast (Δ*ε*) of 0.39 and 0.37, respectively. This indicates both ALD and sputtered W:VO_2_ are of high quality.

### 3.2 Metasurface Design Variations on Small‐Area Meta‐OSR

**FIGURE 2:**
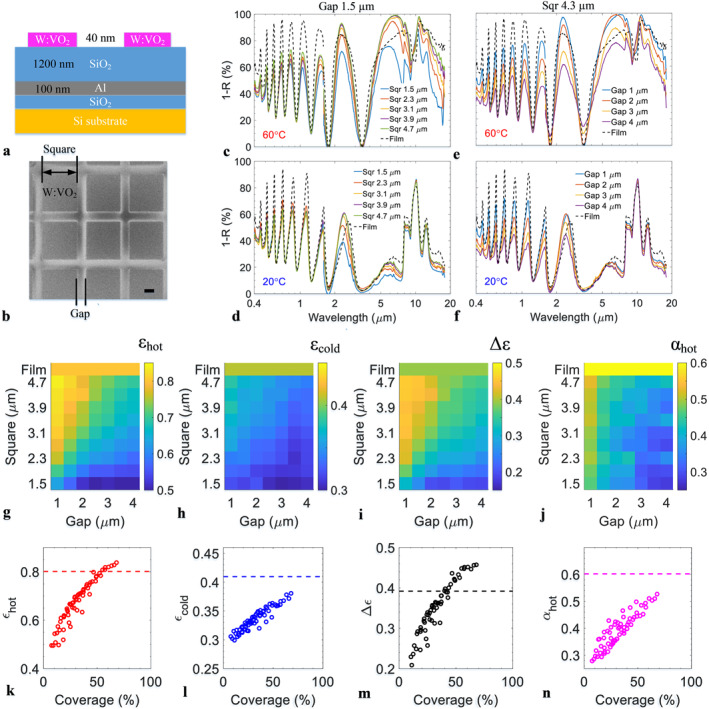
Small‐area W:VO_2_ meta‐OSR on Si substrate for feature size and gap optimization. (a) Cross‐section view schematics of the meta‐OSR without LESR, (b) SEM image of the metasurface with scale bar of 1 μm, (c, d) experimental absorption spectra of meta‐OSRs with various feature size from 1.5 to 4.7 μm at a fixed gap size of 1.5 μm, measured at 60°C (c) and 20°C (d), (e, f) experimental absorption spectra of meta‐OSRs with various gap size from 1 to 4 μm at a fixed feature size of 4.3 μm, measured at 60°C (e) and 20°C (f), (g–j) experimental maps against feature size and gap for hot‐state emissivity, *ε*_hot_ (g), cold‐state emissivity, *ε*_cold_ (h), emissivity contrast, Δ*ε* (i), and hot‐state solar absorption, *α*_hot_ (j) for the fabricated meta‐OSR devices, and (k–n) their relations (*ε*_hot,_ *ε*_cold,_ Δ*ε* and *α*_hot_) as a function of W:VO_2_ coverage, respectively. Dashed lines represent film results.

To explore the optimal metasurface dimensions for the smart meta‐OSR, patterned ALD‐grown W:VO_2_ OSRs were fabricated on a silicon substrate using e‐beam lithography (EBL). The meta‐OSR stack structure contains W:VO_2_ features with square shape and gap between the features as schematically in Figure 2a. Here, the SiO_2_ between the aluminium and silicon was adopted for its better adhesion with the aluminum layer. The square features are varied from 1.5 to 4.7 μm in width in steps of 0.4 μm with gaps ranging from 1 to 4 μm in steps of 0.5 μm. Figure 2b shows a SEM image of a typical W:VO_2_ metasurface array, with a feature size of 1.9 μm and gap of 1 μm. The W:VO_2_ features and gaps are well‐defined with a measured dimension of 1.84 and 0.98 μm, respectively.

Experimental absorption spectra of the fabricated meta‐OSRs are presented in Figure 2c–f for spectral range from 0.4 to 20 μm covering visible, NIR and IR, measured in the hot state (60°C, Figure 2c,e) and cold state (20°C, Figure 2d,f), with results for the unpatterned thin‐film OSRs shown for comparison. Large absorption fringes are present in the visible and near‐IR part of the spectra in both hot and cold states and are the result of Fabry‐Pérot interference in the absorbing stack. Compared to the unpatterned thin‐films (dashed lines), the solar absorption of the metasurface is reduced proportional to the W:VO_2_ coverage. Figure 2c,e shows hot‐state spectra of various square feature size at a fixed gap size of 1.5 μm (c) and for different gaps at a fixed feature size of 4.3 μm (e). In the hot state, the metasurface enhances the IR absorption around 5–10 μm wavelength due to the metallic W:VO_2_ surface plasmon resonance effect, which for the larger feature sizes > 2.3 μm and gaps < 3 μm compensates the loss of absorption due to reduced W:VO_2_ coverage. Corresponding results for the cold state are shown in Figure 2d,f, here W:VO_2_ is dielectric over the entire spectral range and has no plasmonic resonance. The remaining absorption peak around 10 μm corresponds to absorption in the SiO_2_ spacer. The cold‐state IR absorption furthermore decreases with feature size and for increasing gap, proportional to the W:VO_2_ coverage. These results are consistent with previous results obtained for undoped VO_2_ meta‐OSR [8].

Figure 2g–j show resulting experimental maps of meta‐OSR IR emissivity and solar absorption as function of feature size and gap size, measured over 63 individual small area (120 × 120 μm^2^) device arrays fabricated using EBL. The IR emissivity and solar absorption (*α*_hot_) are calculated from experimental spectra ranging from 2.5 to 20 μm and from 0.4 to 2.5 μm, respectively, using equations detailed in Supporting Information S1: Supplementary Materials S3. The results for the unpatterned thin‐film OSR are also presented for comparison in the top bar of each panel labelled ‘film’. IR emissivity information can be extracted through its hysteresis plot as schematically presented in Supporting Information S1: Supplementary Materials S4. In the hot state, the IR emissivity (*ε*_hot_) shows a strong dependence on both feature size and gap, the emissivity peaks at 0.85 for the OSR with 4.7 μm feature size and 1 μm gap, this is higher than 0.81 for the film. Numerically simulated results are presented in Supporting Information S1: Figure S6, Supplementary Materials S6 and show the same trends but a slightly smaller optimal feature size of 3.1 μm. In the cold state, IR emissivity (*ε*_cold_) is little affected by feature size with only a weak dependence on gap size with a maximum around 0.37 and reducing to 0.3 towards the bottom right of the map, lower than 0.42 for the film. The IR emissivity contrast (Δ*ε*) shows feature and gap size dependance and peaks at 0.46 for 4.3 μm and gap 1 μm, higher than 0.39 for the film. Solar absorption in the hot state (*α*_hot_) decreases with increasing gap and shows little change with square size, ranging between 0.3 and 0.5, significantly lower than 0.60 for the film. Therefore, a balance between a high IR emissivity contrast and a low solar absorption (hot) can be achieved by choosing certain W:VO_2_ feature and gap size, and this provides the range for the large‐area meta‐OSR demonstrator devices.

To further elaborate the relationship with feature and gap sizes, the IR emissivity and solar absorption are plotted as a function of the W:VO_2_ coverage ratio in Figure 2k–n. For the hot‐state IR emissivity (*ε*_hot_), the curve shows an unambiguous deviation from a linear dependence on the coverage ratio, which is attributed to the plasmonic resonance enhanced absorption when the W:VO_2_ is in the metallic state. In contrast, the cold‐state IR emissivity follows a monotonic trend with the W:VO_2_ coverage, indicating negligible plasmonic absorption enhancement when the W:VO_2_ is in dielectric state. The near‐field electric field and absorption coefficient *Q*_abs_ for two feature sizes (1.5 and 4.7 μm) were simulated for both hot and cold states, and the results are presented in Supporting Information S1: Figure S7, Supplementary Materials S6. In the hot state, the 1.5 μm and 4.7 square features both exhibit a significantly enhanced electric field and absorption coefficient indicating that these effects are present for a range of feature sizes and consistent with a broadband dipolar mode. The contrast in both the near‐field and absorption coefficient between hot and cold states (Supporting Information S1: Figure S7) confirms the interpretation in terms of a plasmonic absorption enhancement, as reported in our previous work [8]. As a combined effect, the IR emissivity contrast Δ*ε* for the metasurface can be improved over the film by an increased *ε*_hot_ and decreased *ε*_cold,_ driven by plasmonic resonance and W:VO_2_ coverage reduction, respectively. The hot‐state solar absorption increases approximately with W:VO_2_ coverage, as the W:VO_2_ has negligible plasmonic resonance in solar spectral band. It is also worth mentioning that there is some offset in *α*_hot_, because the features induced visible scattering and thereby increase solar absorption, as observed in our previous work on AZO meta‐OSR [2].

Complementary to the reflectivity measurements, experimental verification of the thermal dissipation capability using a calorimetric method were performed and the results are presented in Supporting Information S1: Figure S8, Supplementary Materials S7. The calorimetric method directly measures the radiative heat flow between parallel surfaces under conditions where the test sample surface is heated and the known reference surface is cooled [28]. As direct radiative cooling measurements in space, as used in some terrestrial radiative cooling demonstrators, are beyond the scope of the current work, these cryogenic measurements offer a reference benchmark for the broadband radiative heat dissipation capacity of the coatings.

### 3.3 Low Emissivity Solar Reflector (LESR)

**FIGURE 3:**
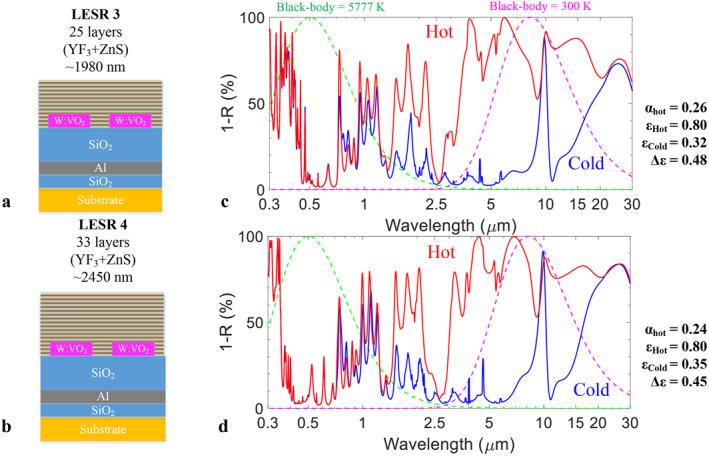
Designs of OSRs including a low emissivity solar reflector (LESR). (a–b) Schematic of LESR 3 (a) and LESR 4 (b), and (c, d) simulated absorption spectra of LESR capped OSRs at hot and low statuses for LESR 3 (c), and LESR 4(d).

To mitigate the visible absorption from the W:VO_2_ film, the LESR capping was designed and implemented on top of the W:VO_2_ metasurface reflector stack. The LESR capping functions to selectively reflect incident solar radiation before it reaches the W:VO_2_ layer and is formed by stack of distributed Bragg reflectors (DBRs). Two LESR designs are schematically presented in Figure 3a,b with an optimization of 3 DBR bands and 4 DBR bands, labelled as LESR 3 and LESR 4, respectively. The LESR is structured as a stack with yttrium fluoride (YF_3_) and zinc sulfide (ZnS) as low and high index materials, which both materials having a low absorption from visible up to thermal infrared. The refractive index values of YF_3_ and ZnS used for numerical modelling are presented in Supporting Information S1: Supplementary Materials S8. LESR 3 consists of 25 layers with a total thickness of about 1980 nm, while LESR 4 has 33 layers and a total thickness of 2450 nm. Whereas in principle the design could be further extended, a thicker LESR is undesired for its influence on foil flexibility.

Figure 3c presents the simulated absorption spectra of the VO_2_ meta‐OSR with LESR 3, for W:VO_2_ feature size of 2.7 μm and gap of 1 μm. The black‐body radiation spectra for the Sun and the 300 K thermal environment are also plotted for reference. The periodic Fabry‐Pérot interference fringes seen for the bare OSR stack are replaced by a more complex transmission function, where the absorption is significantly suppressed in a band around the peak of the solar spectrum around 0.5 μm in both hot and cold states. A significant contrast in the IR absorption is still preserved between the hot and cold states. For the meta‐OSR with LESR 3 capping, we obtain an *α*_hot_ of 0.26, *ε*_hot_ of 0.80 and Δ*ε* of 0.48, indicating that the LESR 3 reduces solar absorption but maintaining IR emissivity contrast. For the meta‐OSR with LESR 4 capping, a similar trend is seen but with a broader visible absorption suppression around 0.5 μm. This results in *α*_hot_ of 0.24, *ε*_hot_ of 0.80 and Δ*ε* of 0.45. Since hot‐state solar absorption is the main concern, the thicker LESR is needed to reduce *α*_hot_ below 0.25. However, the emissivity contrast (Δ*ε*) is seen to decrease with the LESR thickness. Therefore, the thicker LESR 4 is chosen as a trade‐off of low *α*_hot_ and high Δ*ε*.

### 3.4 Large‐Scale W:VO_2_ Meta‐OSR With LESR

**FIGURE 4:**
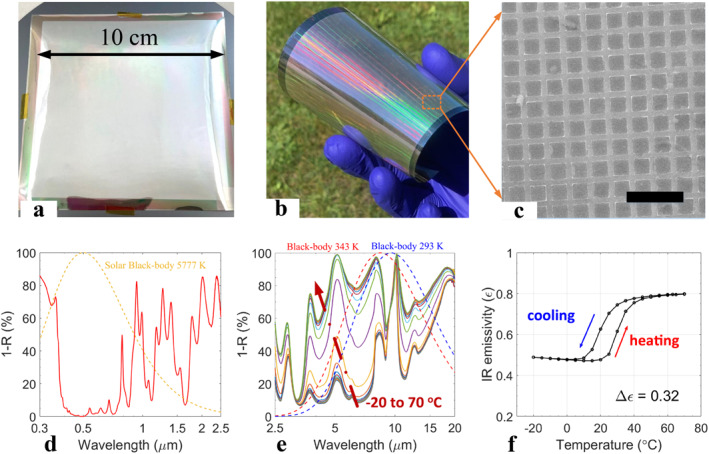
Large‐area (100 × 100 mm^2^) W:VO_2_ meta‐OSR on polyimide foil with LESR cap. (a) Meta‐OSR on polyimide foil mounted on 150 mm Si substrate, (b) outdoor photograph of unmounted meta‐OSR foil, (c) SEM image of the W:VO_2_ metasurface with scale bar of 10 μm, (d) experimental UV/Vis/NIR reflection spectra of the large‐area meta‐OSR with LESR, (e) IR reflection spectra of the meta‐OSR measured at various temperatures, and (f) hysteresis of IR emissivity.

Following the dimensional and LESR optimizations, the full design of ‘smart’ meta‐OSR was fabricated on a polyimide substrate (Dupont Kapton FPC 3 mil). Figure 4a shows a photograph of the fabricated W:VO_2_ meta‐OSR demonstrator device with dimensions of 10 × 10 cm^2^, mounted on a 150 mm Si substrate for processing and handling. All layers, including the sputtered W:VO_2_, were grown using the technology developed by Consorzio C.R.E.O. The central area of about 90 × 90 mm^2^ was patterned as metasurface using nanoimprint lithography by NIL Technology. The full stack structure is schematically shown in Figure 4a (bottom) and the W:VO_2_ array is designed as 2.7 and 1 μm for feature and gap, respectively. Figure 4b shows the photograph of the meta‐OSR when unmounted from the Si substrate as a shiny flexible foil. The SEM micrograph of the W:VO_2_ metasurface before LESR deposition is presented in Figure 4c. The array of W:VO_2_ features are well‐defined as squares, indicating a high‐quality nanoimprint patterning process.

The fabricated meta‐OSR with LESR capping was optically characterized, the reflectivity spectra are presented in Figure 4d,e for the solar radiation band (Vis/NIR) and IR band, respectively. Compared with the experimental spectra of meta‐OSR without LESR, solar absorption is significantly suppressed in the range from 400 to 900 nm wavelength. The solar absorption (*α*_hot_) is calculated to be 0.24, which is significantly improved from the value of 0.41 without LESR for the same device geometry in Figure 2j. Temperature‐dependent IR absorption spectra were measured using FTIR at temperature ranging from −20°C to +70°C as shown in Figure 4e. Compared to the device without LESR, the spectra show much less temperature dependence towards longer wavelengths above 15 μm. This agrees with the numerical simulations and is attributed mainly to the emissivity of the LESR in the long‐wave IR range. The IR emissivity (*ε*) is calculated and plotted in Figure 4f as a function of temperature. The heating and cooling transition temperatures are extracted as 25 and 20°C, respectively, with a hot‐state IR emissivity *ε*_hot_ of 0.80 and IR emissivity contrast Δ*ε* of 0.32.

### 3.5 Space Validation Tests

As the outer coating of the spacecraft, the fabricated meta‐OSRs are required to be able to sustain the harsh space environment. Therefore, on‐earth space qualification tests were performed on high‐performance small samples (25 × 25 mm^2^ each). The test consists of adhesion test, thermal cycling test under vacuum, radiation tests and humidity test, with the test conditions following European Space Agency (ESA) standards. The detailed information of these tests is given in the Methods section and in Supporting Information S1: Table S1, Supplementary Materials S9, together with sample photos in Supporting Information S1: Figures S10 and S11, Supplementary Materials S10.

After each step, the samples underwent quality control through a visual inspection and optical characterizations. Table 1 summarizes the main functional parameters at each stage for a representative sample out of a collection of 17 demonstrator devices. The functional definitions are elaborated in Supporting Information S1: Supplementary Materials S4 and Δ*T*_MIT_ is defined as the hysteresis between heating and cooling transition temperatures. The demonstrator devices deliver properties that remain essentially unchanged after the entire sequence from thermal vacuum cycling (TVAC) to irradiation with protons to humidity. From a mechanical point of view, tests on large area samples demonstrate excellent adhesion–adhesion rate > 4B according to ASTM D3359—after TVAC sequences, and bending, with photos available in Supporting Information S1: Figures S10 and S11, Supplementary Materials S10. The bending test included cycles with the coating in contact with the roller, that is on the concave face of the foil with rollers down to 4 mm. No damage was observed to the coating after this process, showing the excellent properties of the meta‐OSR with LESR stack.

**TABLE 1:** Performance of a typical meta‐OSR at each stage of the space validation test.

Stage	*α*_hot_	*ε*_hot_	Δ*ε*	*T*_MIT_ (°C)	Δ*T*_MIT_ (°C)
Before test (BoL)	0.22	0.78	0.33	30	10
Thermal test	0.24	0.74	0.35	30	10
Radiation test	0.23	0.75	0.33	30	10
Humidity test (EoT)	0.22	0.75	0.33	30	10

## 4 Discussion

Vanadium dioxide (VO_2_) based space thermal management solutions have been extensively investigated in recent studies. Although VO_2_ reflectors exhibit desirable infrared emissivity contrast for tunable thermal control, they still face several technical challenges including the critical improvements (i)–(iv) as addressed in our work. Table 2 summarizes recent works on VO_2_‐based reflectors, including our previous work [8], which demonstrated patterned VO_2_ structures with enhanced solar absorption on silicon. In other studies, the solar absorption reached as high as 0.8 for a three‐layer Salisbury screen structure [17]. These works demonstrated excellent IR emissivity contrast, but the solar absorption issue has not been sufficiently addressed. These VO_2_ OSRs were formed on rigid silicon substrates with a VO_2_ process temperature incompatible with polyimide substrates.

**TABLE 2:** Performance of VO_2_‐based reflectors with tunable emissivity.

Year	Method/W:VO_2_ deposition	Structure	Space test	Substrate/litho, sample area	*α*_hot_	Δ*ε*	*ε*_hot_	*ε*_cold_	*T*_MIT_ (°C)	Dep. *T* (°C)	Refs
2025	Exp. Sputter Sim.	LESR/meta‐W:VO_2_/SiO_2_/Al	Yes	Polyimide/NIL, 10 × 10 cm^2^	0.22	0.33	0.80	0.47	30	375	This work
					0.24	0.45	0.80	0.35			
2022	Exp. ALD	Meta‐W:VO_2_/SiO_2_/Al	No	Polyimide/photolith, 2.5 × 2.5 cm^2^	—	0.35	0.72	0.24	28	375	[27]
2022	Exp. Sputter	VO_2_/SiO_2_/Ag	No	Silicon/planar, area unknown	0.80	0.52	0.59	0.07	68	400	[17]
2021	Exp. PLD	Meta‐W:VO_2_/BaF_2_/Ag	No	Polyimide/photolith, 2 × 2 cm^2^	0.25	0.45^a^	0.68^a^	0.23^a^	22	500	[24]
2018	Exp. Sputter	Meta‐VO_2_/SiO_2_/Al	No	Silicon/EBL, 2 × 2 cm^2^	0.46	0.48	0.64	0.32	68	375	[8]
2024	Sim.	DBR/VO_2_/BaF_2_/Al	—	—	0.12	0.83	0.92	0.09	—	—	[29]
2023	Sim.	DBR/VO_2_/BaF_2_/Ag	—	—	0.05	0.64	0.73	0.09	—	—	[30]
2023	Sim.	VO_2_/SiO_2_/Al_2_O_3_/Ag	—	—	0.38	0.78	—	—	—	—	[19]

Abbreviations: ALD, atomic layer deposition; DBR, distributed Bragg reflector; EBL, e‐beam lithography; LESR, low emissivity solar reflector; NIL, nanoimprint lithography; PLD, pulsed laser deposition.

^a^ Based on the best estimation in available spectra range, and optimized for atmosphere windows.

A recent work [24] achieved significant progress using a W:VO_2_ metasurface reflector, which exhibited low solar absorption and high IR emissivity in the terrestrial radiative cooling window range of 8–14 μm. As a proof‐of‐concept work, it overcame the thermal processing constraint by forming W:VO_2_ on silicon substrate by pulsed laser deposition (PLD) and 500°C anneal, followed by transfer onto a flexible substrate. This work demonstrated excellent performance in both high IR emissivity contrast, low solar absorption and room‐temperature transition. However, it is a small‐area (20 mm × 20 mm) demonstrator and its PLD growth is unsuitable for scaling up, as well as its manufacturing complexity involving substrate transferring. It is also worth mentioning that this design was optimized for terrestrial applications towards atmosphere transmission window, which is distinctive from space application environment and considerations.

In comparison, the present work employs large‐area growth techniques—atomic layer deposition and sputtering/oxidation—combined with low temperature processing that is directly compatible with space‐grade polyimide substrates. Our optimized low‐temperature VO_2_ films have been demonstrated at formation temperatures as low as 300°C [31]. We also adopted the nanoimprint lithography technique to manufacture 10 cm × 10 cm large‐area metasurfaces over polyimide substrate directly. In addition, we adopted a low emissivity solar reflector (LESR) to minimize solar absorption. Importantly, our W:VO_2_ reflectors have successfully passed several space qualification tests, making them ready for space environment verification.

The frequency‐selective DBR solutions for the visible solar band have also been reported in recent simulation studies, which suggest further performance improvements could be achieved through optimization of the dielectric spacer material and selective reflector design. Nonetheless, new challenges remain if adopting the design in these simulation works, such as the fabrication of high‐quality ultrathin W:VO_2_ and BaF_2_ formation.

According to thermal analysis results provided by Thales Alenia Space (*F. Tessarin* et al.*, private communication*, 2024), the proposed smart OSR was evaluated under conditions of low Earth orbit (LEO), medium Earth orbit (MEO) and geostationary orbit (GEO) mission conditions. Compared with a conventional OSR (*ε* = 0.8), our smart meta‐OSR with Δ*ε* = 0.3 achieves approximately 40% power saving in heater usage during the cold phase. The savings can be projected to increase to 50% and 60% with further enhancements to Δ*ε* = 0.35 and 0.4, respectively. Therefore, the proposed smart meta‐OSR offers significant system‐level advantages, including reducing mass, simplified thermal control architecture, and decreased battery volume. Further reduction in solar absorption *α* to below 0.2 is a priority in future development. The use of nano‐imprint lithography for large‐area patterning of sub‐micrometre features holds promise for scaling up the meta‐OSR technology.

## 5 Conclusions

In conclusion, we have demonstrated large‐area W‐doped VO_2_ metasurface optical solar reflectors (meta‐OSRs) fabricated on polyimide substrates. The solar absorption and infrared emissivity contrast were optimized by experimentally investigating small‐area samples with varying feature dimensions. Using a nanoimprint lithography, we achieved 10 × 10 cm^2^ large‐area W:VO_2_ meta‐OSRs directly on polyimide. With a low emissivity solar reflector (LESR) capping layer, the W:VO_2_ meta‐OSR exhibited a solar absorption (*α*) of 0.22, high‐temperature emissivity (*ε*_hot_) of 0.8, infrared emissivity contrast (Δ*ε*) of 0.33 and transition temperature (*T*_MIT_) of about 30°C. In addition, the fabricated meta‐OSR successfully passed various space qualification tests, including adhesion, bending, thermal cycling, proton and electron irradiation and humidity, showing negligible performance degradation. These results indicate strong potential for further space environmental verification in orbit.

## 6 Methods

### 6.1 Small‐Area Meta‐OSR Fabrication

The small‐area meta‐OSRs were fabricated on SiO_2_ coated Si substrate. A 100 nm aluminum was grown on the SiO_2_ layer using sputtering. Subsequently, a 1200 nm SiO_2_ were deposited on Al as a spacer using Si target and O_2_ plasma in a Bühler Helios sputtering system. Then, a 40 nm W:VO_2_ was grown on the spacer SiO_2_ layer using Savanah S200 ALD system. The W:VO_2_ ALD process was carried out at 200°C using Tetrakis (ethylmethylamino) vanadium (IV) (TEMAV) 98% and Tungsten hexacarbonyl (W(CO)_6_) 99% (both from Strem Chemicals) and deionized water as oxidizer. The full process details are available in our previously published work [27]. The growth ratio of tungsten cycle to vanadium cycle is 0.8, giving about 1.5 at. % of tungsten [27]. The deposited W:VO_2_ was annealed in Oxford Instruments Agile NanoFab system at 400°C for 2 h at oxygen partial pressure of 1 Torr. The W:VO_2_ metasurface was fabricated through electron beam lithography (JEOL JBX‐9300FS) using ZEP520A and Oxford Instruments IonFab 300 Plus ion beam system. The resist was stripped using a cycled low temperature O_2_ ICP plasma process to avoid further oxidizing the W:VO_2_.

### 6.2 Large‐Area Meta‐OSR on Flexible Substrate With LESR

The large‐scale Smart‐OSRs were fabricated on polyimide (Dupont Kapton FPC 3 mil), mounted on 6‐inch carrier wafers. First, a metallic Al layer and a 1100 nm SiO_2_ as the dielectric spacer were deposited by RF sputtering, using a deposition plant equipped with two 8″ rounded magnetron cathodes. Then, the photonic pattern was drawn by nano‐imprint lithography followed by the sputter deposition of a W‐doped vanadium layer, and chemical lift‐off. A fast post annealing process was performed to convert W‐doped vanadium structures into W:VO_2_ features in thickness of 30 nm. Finally, the uppermost LESR consisting of ZnS and YF_3_ was grown by ion‐beam evaporation. Extra dielectric and conductive layers were also grown on top of the LESR as final passivation and protection.

### 6.3 Optical Characterizations

The metasurface was characterized by UV–Vis systems and FTIR systems. For small‐area ALD W:VO_2_ samples, the UV–Vis and IR measurements were done using a home‐made UV–Vis with a heater and Thermo‐Nicolet Nexus 670 FTIR with a Linkam THMS 600 stage. For the FTIR For large‐area sputtered W:VO_2_ reflectors, the UV–Vis and IR measurements were done using Perkin‐Elmer Lambda 950 UV–VIS with 150 mm integration sphere, and Perkin‐Elmer Frontier FTIR, respectively. Solar absorption and emissivity values were calculated following standard ECSS‐Q‐ST‐70‐09C, even if with small waiver for *ε*N.

### 6.4 Space Qualification Testing

Tests were done in accordance with ASTM D 3359 ‘Standard Test Methods for Measuring Adhesion by Tape Test’, ECSS‐Q‐ST‐70‐04C ‘Thermal testing for the evaluation of space materials, processes, mechanical parts and assemblies (15 November 2008)’, ECSS‐Q‐ST‐70‐06C ‘Particle and UV radiation testing for space materials (31 July 2008)’. The full test sequence was performed on a total of 17 samples of size 25 × 25 mm^2^ and active area ≥ 10 × 10 mm^2^. All selected samples were fully described at beginning of life. *Thermal tests*: the demonstrators underwent 105 cycles of thermal vacuum (TVAC) between −70 and +180°C (MAPRad, Italy). Surviving samples were tested for additional 8 TVAC cycles between −180 and +180°C (AAC, Austria). Samples were then tested for thermal ageing for 350 h at 90°C at a pressure of 3 × 10^−7^ mbar. *Radiation tests:* proton radiation tests were performed at Ion Beam Centre (IBC) of Helmholtz Zentrum Dresden Rossendorf (HZDR), Germany. Samples were exposed to two types of protons: low energy: 50 keV at 1.10 × 10^15^ cm^−2^ fluence and 5.00 × 10^11^ cm^−2^s^−1^ flux and high energy: 250 keV at 2.10 × 10^14^ cm^−2^ fluence and 5.00 × 10^10^ cm^−2^s^−1^ flux. One sample was also tested against electron irradiation: 200 keV at 1.35 × 10^16^ cm^−2^ fluence and 5 × 10^11^ cm^−2^s^−1^ flux.

### 6.5 Numerical Modelling

The simulations of the meta‐OSR with LESR were done using the finite difference time domain (FDTD) method implemented in Lumerical software. The near‐field mapping was simulated through COMSOL finite elemental modelling, with linearly polarized incident light and square corner edges rounded by 20 nm radius of curvature.

## Author Contributions

**Mirko Simeoni:** conceptualization, investigation, writing – original draft, writing – review and editing, formal analysis, methodology, data curation. **Kai Sun:** conceptualization, investigation, writing – original draft, visualization, methodology, writing – review and editing, formal analysis, data curation. **Alessandro Urbani:** investigation, writing – review and editing, methodology. **Ioannis Zeimpekis:** writing – review and editing, investigation, formal analysis. **Ilja Czolkos:** investigation, validation, writing – review and editing, formal analysis, data curation. **Lars Kildebro:** investigation, writing – review and editing, formal analysis, data curation, validation. **Matteo Gaspari:** investigation, writing – review and editing, validation, formal analysis, data curation. **Giovanni Bartolini:** investigation, methodology, formal analysis. **Behcet Alpat:** investigation, funding acquisition, writing – review and editing, methodology, project administration, data curation, supervision, formal analysis, validation. **Jiri Frolec:** methodology, formal analysis, investigation, writing – review and editing. **Tomas Kralik:** investigation, methodology, writing – review and editing, formal analysis. **Cornelis H. (Kees) de Groot:** conceptualization, funding acquisition, writing – review and editing, methodology, supervision. **Otto L. Muskens:** conceptualization, investigation, funding acquisition, writing – original draft, methodology, writing – review and editing, project administration, supervision, resources, visualization. **Sandro Mengali:** conceptualization, investigation, funding acquisition, writing – review and editing, writing – original draft, methodology, validation, formal analysis, project administration, supervision, resources, data curation.

## Conflicts of Interest

The authors declare potential commercial interest in the smart meta‐OSR technology presented in this paper.

## Supporting information


Supporting Information S1

## Data Availability

The data that support the findings of this study are openly available in the University of Southampton research repository at https://doi.org/10.5258/SOTON/D3707.
