# Supplementary material for: Smart and Flexible Optical Solar Reflectors for Passive Radiative Cooling Regulation in Space Using a W:VO2 Metasurface
Source: Nanophotonics. 2026 Jan 28;15(3):e70011. doi: 10.1002/nap2.70011 (PMC12964990; doi:10.1002/nap2.70011)
Supplement: Supplementary file 1 — Supporting Information S1 [file NAP2-15-e70011-s001.docx]

**Supplementary Materials for Smart and Flexible Optical Solar Reflectors for Passive Radiative Cooling Regulation in Space using a W:VO_2_ Metasurface**

Mirko Simeoni^1, †^, Kai Sun^2, †^, Alessandro Urbani^1^, Ioannis Zeimpekis^3^, Ilja Czolkos^4^, Lars Kildebro^4^, Matteo Gaspari^1^, Giovanni Bartolini^5^, Behcet Alpat^5^, Jiri Frolec^6^, Tomas Kralik^6^, Cornelis H. (Kees) de Groot^3^, Otto L. Muskens^2,^* and Sandro Mengali^1,^*

^1^ Consorzio C.R.E.O., L’Aquila, 1-67100, Italy

^2^ Astronomy and Physics, Faculty of Engineering and Physical Sciences, University of Southampton, Southampton, SO17 1BJ, UK

^3^ Electronics and Computer Science, Faculty of Engineering and Physical Sciences, University of Southampton, Southampton, SO17 1BJ, UK

^4^ NIL Technology, Diplomvej 381, 2800 Kongens Lyngby, Denmark

^5^ BEAMIDE S.r.l, Perugia, 06127, Italy

^6^ Institute of Scientific Instruments of the CAS, Brno, 612 00, Czech Republic

^†^ MS and KS contributed equally to this work.

^*^ Corresponding author: [O.Muskens@soton.ac.uk](mailto:O.Muskens@soton.ac.uk) and [Sandro.mengali@consorziocreo.it](mailto:Sandro.mengali@consorziocreo.it)

# Raman Spectroscopy of the W:VO_2_

The W:VO_2_ films fabricated by either sputtering and ALD were characterized by Raman spectroscopy to ensure its crystallinity as monoclinic. The measurements was done using Renishaw inVia Raman system using 532 nm laser at room temperature. Due to its near-room temperature transition, W:VO_2_ spectra were found to be noisy over VO_2_ as seen in our previous work.^1^ The twin peaks at 192 cm^-1^ and 223 cm^-1^ and the 610 peak cm^-1^ are consistent with VO2 and thus confirm their monoclinic crystallinity.


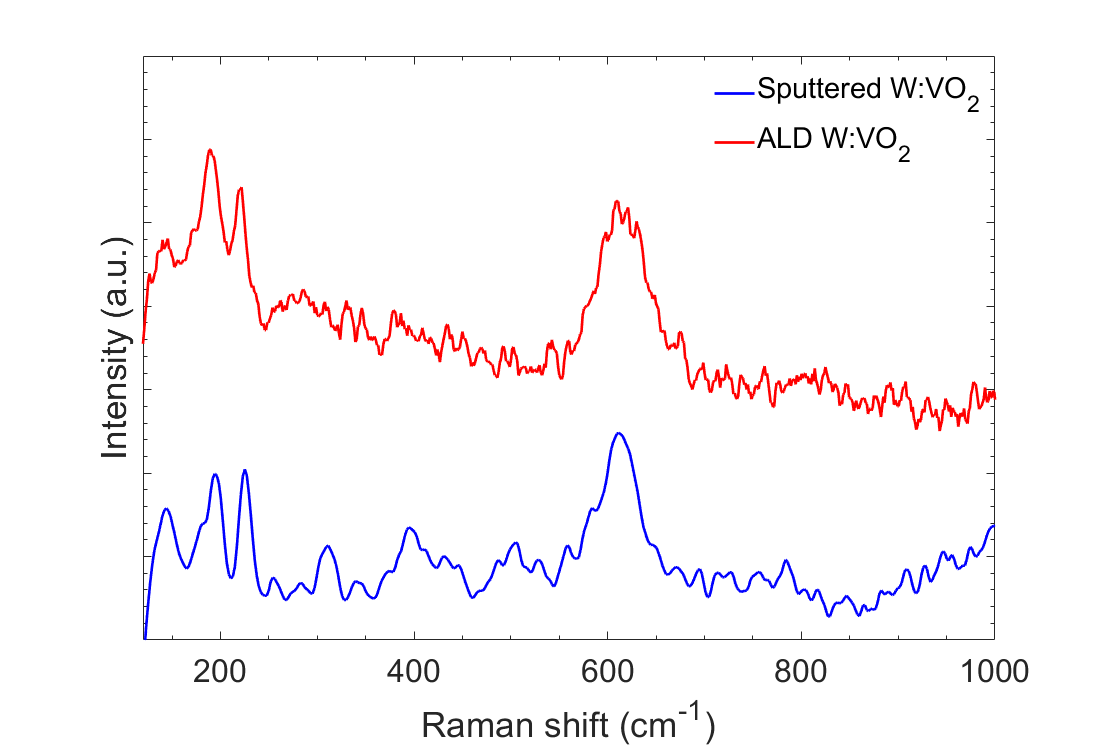


Figure S1 Raman spectroscopy for W-doped VO_2_ films by sputtering and ALD.

# FTIR Measurement at different temperatures

In this work, the FTIR measurements were done using two FTIR systems at Southampton University (Figure S2) and C.R.E.O. (Figure S3). Southampton University setup involves a Thermo-Nicolet Nexus 670 with Continuum microscope using a X15 optical objective, MCT-A detector, KBr detector and IR source. The sample temperature was controlled by a Linkam THMS600 stage with a ZnS window, equipping with liquid nitrogen cooling. The reflection measurements were normalized using an aluminum mirror. C.R.E.O. setup involves a Perkin-Elmer Frontier FT-IR spectrophotometer equipped with a homemade box with a sample holder cooling through liquid N_2_ flux with a dewar on side.

Figure S2 FTIR setup (Southampton University) with Linkam heating stage for temperature varied characterizations.


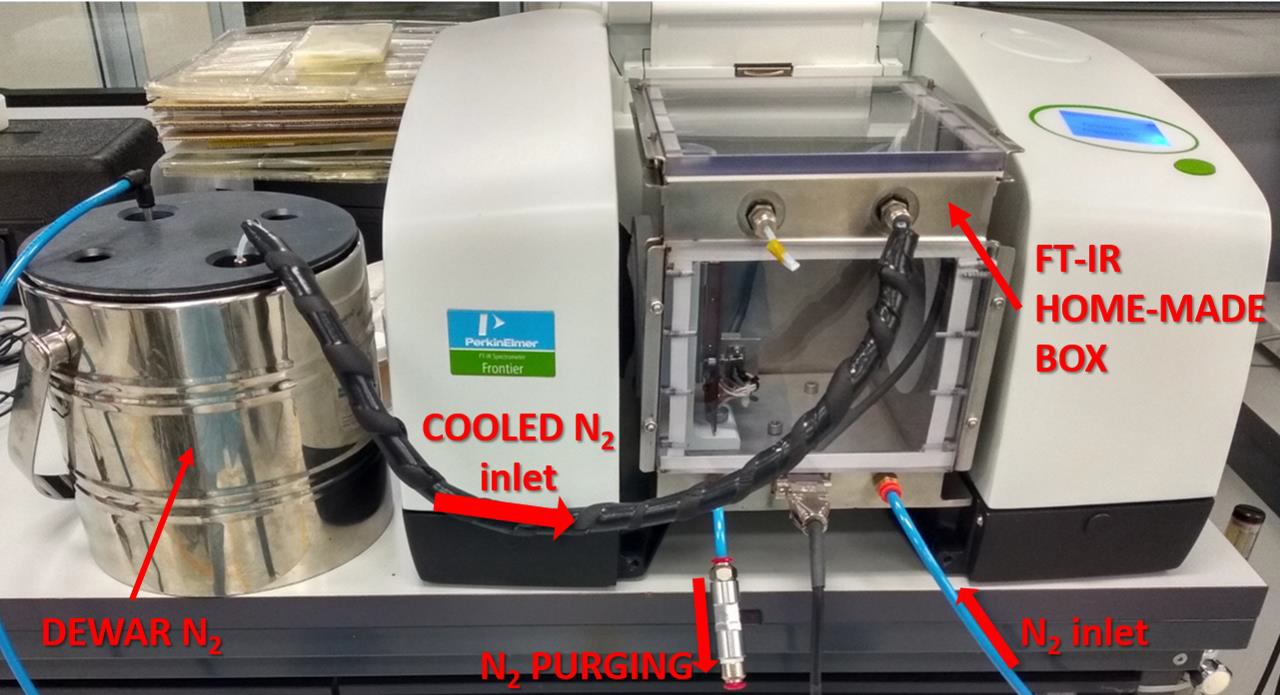


Figure S3 Perkin-Elmer FTIR setup (CREO) with homemade heating/cooling stage.

# Solar absorption and IR Emissivity Calculation

For opaque samples – as it is the case for the present work – solar absorption and infrared emittance can be calculated starting from reflectance spectra, in the UV-VIS-NIR, and TIR spectral range, respectively. The spectral responses of DUT (Device Under Test) shall be weighted with the black-body irradiation at the right temperature as below.

The spectral radiation of a black body at temperature *T* can be given in terms of wavelength:

$B\left( T,\lambda\right)=\frac{2hc^{2}}{\lambda^{5}}\frac{1}{exp\left( {hc}/{\lambda kT} \right)-1}$ *Eq.1*

where *B*(*T, λ*) is black body radiation in SI unit of *B*(*T, λ*) is W·sr^-1^·m^-3^, *h* is Plank constant of 6.626×10^-34^ J.s, *c* is light speed in vacuum 3×10^8^ m/s, *k* is Boltzmann constant of 1.38×10 ^-23^ J/K and *λ* is the wavelength.

According to ECSS-Q-ST-09C, Solar absorption (α) at temperature T_S_ shall be calculated in wavelength of 0.25 to 2.5 µm (covering about 96 % of the total solar energy):

$a\left( T_{s} \right)=1- \frac{\int_{0.25 \mu m}^{2.5 \mu m} A\left( \lambda, T_{s} \right)B\left( \lambda, T_{sun} \right)d\lambda}{\int_{0.25 \mu m}^{2.5 \mu m} B\left( \lambda, T_{sun} \right)d\lambda}$ *Eq.2*

where *T*_s_ is the DUT temperature , *T*_sun_ is the Sun’s surface temperature (5777 K), *A*(λ, *T*_s_) is the absorption spectra at temperature of *T*_s_, *B*(λ, *T*_sun_) is the solar black-body radiation.

According to ECSS-Q-ST-09C, infrared emissivity (ε) can be calculated in wavelength of 3 to 20 µm:

$\varepsilon(T_{s})=\frac{\int_{3 \mu m}^{20 \mu m} A\left( \lambda, T_{s} \right)B\left( \lambda, T_{s} \right)d\lambda}{\int_{3 \mu m}^{20 \mu m} B\left( \lambda, T_{s} \right)d\lambda}$ *Eq.2*

where λ is the wavelength, *T*_s_ is the temperature on the optical reflector surface, *A*(λ, *T*_s_) is the spectral absorbance after 100% reference correction at temperature *T*_s_, *B*(λ, *T*_s_) is the black-body spectrum at the same temperature. In terms of solar absorption and IR emissivity integration range, they were tailored to available measured spectra.

Since W:VO_2_ is a phase-transition material, and – as a consequence – the DUT (Device Under Test) change its spectral response with the temperature – both in the UV-VIS-NIR and IR range – , solar absorption and IR emissivity at the two the statuses are referred as α_cold_ and α_hot_, and ε_cold_ and ε_hot_, when at dielectric and metallic status, respectively.

# IR emissivity hysteresis and transition temperature extraction

Figure S4a shows the IR emissivity hysteresis curve and the definitions of all relevant terms, ε_hot_, ε_cold_ , and Δε. Figure S4b shows the differential plot of the IR emissivity and its peaks correspond to the transition temperature (*T*_MIT_). Technically, there are two transition temperatures for heating and cooling, but for clarity and consistence, the heating transition temperature is noted as *T*_MIT._

Figure S4 (a) IR emissivity (ε) Hysteresis and (b) differential of the IR emissivity, both as a function of T.

# Infrared response of W:VO_2_ reflectors by ALD and sputtering

Figure S5 shows absorption spectra of unpatterned W:VO_2_ reflectors, formed by ALD (a) and by sputtering (b). The spectra were measured without low emissivity solar reflector (LESR) formation using FTIR over a temperature range for the two films.

(a)

(c)

(b)

(d)

0 to 80 ^o^C

-30 to 60 ^o^C

Figure S5 Planar W:VO_2_ reflector Infrared responses. (a, b) IR absorption spectra against temperature for the heating part of the cycle for ALD W:VO_2_ (a) at 0 ^o^C to 80 ^o^C and sputtered W:VO_2_ (b) at -30 ^o^C to 60 ^o^C, and (c,d) IR emissivity hysteresis.

# Simulations of VO_2_ meta-OSRs without LESR

Figure S6 shows simulated VO_2_ metasurface responses in terms of ε_hot_, ε_cold_, Δε, α_hot_ and α_cold_ as function of square and gap dimensions. The results are based on the numerically simulated spectra of VO_2_ metasurface reflectors without LESR structures.

Figure S6 Numerically simulated metasurface responses against square and gap dimension, in terms of ε_hot_, ε_cold_, Δε, and α_hot_.

Figure S7 shows simulated W:VO_2_ surface near-field (*E*/*E*_in_) and absorption coefficient (*Q*_abs_). The simulations were done using COMSOL with feature corner rounded at 20 nm curvature radius. The simulations were taken at wavelength of 6.5 μm and 16 μm at cold and hot states. The two simulated structures are for 1.5 μm and 4.7 μm square features at gap size of 1 μm.


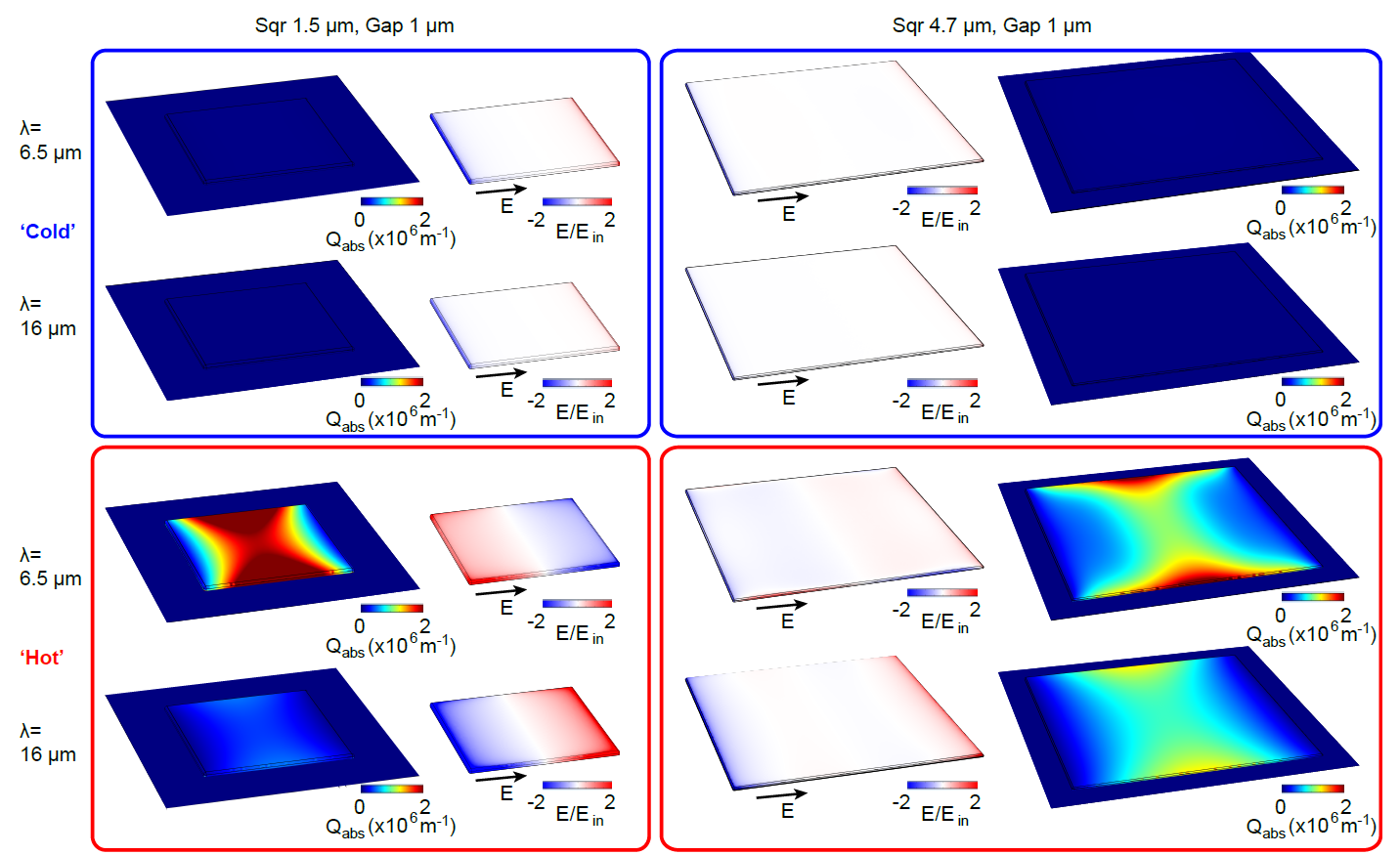


Figure S7 Calculated normal near-field (E/E_in_) at W:VO_2_ surface and absorption coefficient (Q_abs_) at 6.5 μm and 16 μm at cold and hot states.

# Calorimetric Measurement of the OSR without LESR

The calorimetric measurement was performed at the Institute of the Scientific Instruments of the CAS (Czech Republic) following previously published methods.^2^ The measurement was performed in a thermally isolated configuration to measure mutual emissivity between the system radiator (our W:VO_2_ meta-OSR) and absorber (reference black coating), with both temperatures monitored as T_R_ and T_A_, respectively.

The meta-OSR was cut in diameter of 40 mm and mounted onto a copper holder as shown in inset photo of Figure S8a. Unlike the FTIR with a normal emissivity, the Calorimetric measurement takes scattering, angular dependence and entire EM spectrum into consideration. The calculated emissivity is presented in Figure S8a for the cooling down part of the cycle. Due to the specifics of the setup it can only be heated up to a maximum temperature of 47 ^o^C. The full experimental range is plotted in Figure S8a down to 20 K, with a detail of the IR emissivity transition over the 280 K - 320 K (7 °C - 47 °C) range shown in Figure S8b.

Within this range, the IR emissivity decreases from about 0.55 to 0.27, confirming the temperature-adapted IR emissivity of the fabricated W:VO_2_ OSR. Due to the limited temperature range, the calorimetry setup cannot give accurate determination over the full transition and the high-temperature point of the transition cannot be clearly determined. The calorimetry method generally gives lower emittance than spectroscopic measurements which is expected as it weights the far-infrared spectrum above 25 μm wavelength, in which coatings and films tend to be transparent.

Calorimetry offers a direct method of measuring radiative heat efficiency which is complementary to the reflectance method and which is used here as a reference benchmark for an individual sample.

Figure S8 IR emissivity of a typical W:VO_2_ meta-OSR without LESR from Calorimetric measurements, (a) full range and (b) zoom-in around 280-320 K (7-47 °C). The inset is the measurement configurations and photo of 40 mm diameter OSR mounted on copper holder.

# Optical properties for YF_3_ and ZnS

Figure S5 Refractive index of YF3 and ZnS used in LESR simulations.

Figure S9 Refractive index of YF_3_ and ZnS used in LESR simulations.

YF_3_

ZnS

# Space qualification tests

Table S1 Test matrix for the high-performance demonstrators.

| **Test** | **Description** | **Standard** |
| --- | --- | --- |
| 1. Inspection @BoL^(a)^ | visual inspection |  |
| 1. Thermo-Optical @BoL | α, ε_N_(T) | ECSS-Q-ST-70-09C ^(b)^ |
| 1. Adhesion @BoL | adhesion tape test | ASTM D3359 |
| 1. TVAC [-70; +180] °C   Temp. gradient 6±1 °C/min | 105 cycles | ECSS-Q-ST-70-04C |
| 1. TVAC [-180; +180] °C   Temp. Gradient 8±1 °C/min | 8 cycles | ECSS-Q-ST-70-04C |
| 1. Thermal Ageing | 350 hours @90 °C,  @3.0×10^-7^ mbar | ECSS-Q-ST-70-04C |
| 1. Low Energy Protons | H^+^ @50 keV – fluence 1.10×10^15^ cm^-2^, 5×10^11^ cm^-2^s^-1^ flux | ECSS-Q-ST-70-06C |
| 1. High Energy Protons | H^+^ @250 keV – fluence 2.10×10^14^ cm^-2^, flux 5×10^10^ cm^-2^s^-1^ flux | ECSS-Q-ST-70-06C |
| 1. Electron radiation | 200 keV, 1.35×10^16^ cm^-2^ – fluence, 5×10^11^ cm^-2^s^-1^ flux | ECSS-Q-ST-70-06C |
| 1. Humidity (1 day) | 1 day @ T=45±5 °C – RH%= 95±5 |  |
| 1. Inspection @EoT^(c)^ | visual inspection |  |
| 1. Thermo-Optical @EoT | α, ε(T) |  |
| 1. Adhesion @EoT | adhesion tape test |  |
| a) Begin of Life; b) Tailored for the infrared normal emissivity ε calculation starting from specular reflection measurements without integrating sphere; c) End of Test; | | |

# Photos of samples with space qualification tests

Figure S10 Photos of the smart OSR demonstrator at before/after serial tests and adhesion test.

Figure S11 Photo of the smart OSR demonstrator under bending tests.

Reference

(1) Sun, K.; Wheeler, C.; Hillier, J. A.; Ye, S.; Zeimpekis, I.; Urbani, A.; Kalfagiannis, N.; Muskens, O. L.; de Groot, C. H. Room Temperature Phase Transition of W-Doped VO_2_ by Atomic Layer Deposition on 200 mm Si Wafers and Flexible Substrates. *Advanced Optical Materials* **2022**, *10* (23), 2201326. DOI: <https://doi.org/10.1002/adom.202201326>.

(2) Králík, T. ; Musilová, V. ; Hanzelka, P. ; Frolec, J. Method for measurement of emissivity and absorptivity of highly reflective surfaces from 20 K to room temperatures, *Metrologia* **2016**, 53(2), 743. DOI: <https://doi.org/10.1088/0026-1394/53/2/743>.
